# High Expression of FGD3, a Putative Regulator of Cell Morphology and Motility, Is Prognostic of Favorable Outcome in Multiple Cancers

**DOI:** 10.1200/PO.17.00009

**Published:** 2017-10-13

**Authors:** Scooter Willis, Yuliang Sun, Mark Abramovitz, Teng Fei, Brandon Young, Xiaoqian Lin, Min Ni, Justin Achua, Meredith M. Regan, Kathryn P. Gray, Robert Gray, Victoria Wang, Bradley Long, Roswitha Kammler, Joseph A. Sparano, Casey Williams, Lori J. Goldstein, Roberto Salgado, Sherene Loi, Giancarlo Pruneri, Giuseppe Viale, Myles Brown, Brian Leyland-Jones

**Affiliations:** **Scooter Willis**, **Yuliang Sun**, **Mark Abramovitz**, **Brandon Young**, **Xiaoqian Lin**, **Justin Achua**, **Casey Williams**, and **Brian Leyland-Jones**, Avera Cancer Institute, Sioux Falls, SD; **Teng Fei**, **Meredith M. Regan**, **Kathryn P. Gray**, **Robert Gray**, **Victoria Wang**, and **Myles Brown**, Dana-Farber Cancer Institute, Boston, MA; **Min Ni**, Children’s Medical Center Research Institute, University of Texas Southwestern Medical Center, Dallas, TX; **Bradley Long**, Molecular Core, Scripps Florida, Jupiter, FL; **Roswitha Kammler**, International Breast Cancer Study Group, Bern, Switzerland; **Joseph A. Sparano**, Montefiore Medical Center, Bronx, NY; **Lori J. Goldstein**, Fox Chase Cancer Center, Philadelphia, PA; **Roberto Salgado**, Breast Cancer Translational Research Laboratory/Institut Jules Bordet, Brussels, Belgium; **Sherene Loi**, Peter MacCallum Cancer Centre, East Melbourne, VIC, Australia; and **Giancarlo Pruneri** and **Giuseppe Viale**, European Institute of Oncology, University of Milan, Milan, Italy.

## Abstract

**Purpose:**

Identification of single-gene biomarkers that are prognostic of outcome can shed new insights on the molecular mechanisms that drive breast cancer and other cancers.

**Methods:**

Exploratory analysis of 20,464 single-gene messenger RNAs (mRNAs) in the Molecular Taxonomy of Breast Cancer International Consortium (METABRIC) discovery cohort indicates that low expression of *FGD3* mRNA is prognostic for poor outcome. Prognostic significance of faciogenital dysplasia 3 (FGD3), SUSD3, and other single-gene proliferation markers was evaluated in breast cancer and The Cancer Genome Atlas (TCGA) cohorts.

**Results:**

A meta-analysis of Cox regression of *FGD3* mRNA as a continuous variable for overall survival of estrogen receptor (ER)–positive samples in METABRIC discovery, METABRIC validation, TCGA breast cancer, and Combination Chemotherapy in Treating Women With Breast Cancer (E2197) cohorts resulted in a combined hazard ratio (HR) of 0.69 (95% CI, 0.63 to 0.75), indicating better outcome with high expression. In the ER-negative samples, the combined meta-analysis HR was 0.72 (95% CI, 0.63 to 0.82), suggesting that FGD3 is prognostic regardless of ER status. The potential of *FGD3* as a biomarker for freedom from recurrence was evaluated in the Breast International Group 1-98 (BIG 1-98; Letrozole or Tamoxifen in Treating Postmenopausal Women With Breast Cancer) study (HR, 0.85; 95% CI, 0.76 to 0.93) for breast cancer–free interval. In the Hungarian Academy of Science (HAS) breast cancer cohort, splitting on the median had an HR of 0.49 (95% CI, 0.42 to 0.58) for recurrence-free survival. A comparison of the Stouffer *P* value in five ER-positive cohorts showed that FGD3 (*P* = 3.8^E-14^) outperformed MKI67 (*P* = 1.06^E-8^) and AURKA (*P* = 2.61^E-5^). A comparison of the Stouffer *P* value in four ER-negative cohorts showed that FGD3 (*P* = 3.88^E-5^) outperformed MKI67 (*P* = .477) and AURKA (*P* = .820).

**Conclusion:**

*FGD3* was previously shown to inhibit cell migration. FGD3 mRNA is regulated by *ESR1* and is associated with favorable outcome in six distinct breast cancer cohorts and four TCGA cancer cohorts. This suggests that FGD3 is an important clinical biomarker.

## INTRODUCTION

An increasing collection of breast cancer cohorts have been molecularly profiled on Affymetrix and Illumina platforms, so it is now feasible to conduct an exploratory analysis to identify single-gene biomarkers in which messenger RNA (mRNA) expression is prognostic of outcome.^1^ By limiting the exploratory analysis to a single gene, we intended to identify novel gene(s) that might provide insight into biological mechanisms that drive breast cancer metastasis.^2^ The starting point for this analysis was the Molecular Taxonomy of Breast Cancer International Consortium (METABRIC) data set, which contains clinical traits, expression data, copy number variation profiles, and single nucleotide polymorphism genotypes derived from breast tumors collected from participants in the METABRIC trial.^3^ We also used the Genomic Data Commons, which incorporates The Cancer Genome Atlas (TCGA) cancer cohorts and currently spans 21 cancer types suitable for survival analysis. This type of analysis could lead to the discovery of prognostic biomarkers that behave as oncogenic drivers and could potentially be novel therapeutic targets.

In this study, we identified *FGD3* mRNA expression as a putative biomarker prognostic of outcome in the METABRIC cohort. *FGD3* and *SUSD3*^4^ cell motility genes were compared as a single-gene biomarker with proliferation genes *MKI67*,^5,6^ *AURKA*,^7-10^ and *PCNA*^11,12^ in six distinct breast cancer cohorts and as a pan-cancer biomarker in TCGA cancer cohorts. FGD3 protein expression was evaluated in breast cancer tissue microarrays (TMAs) as an indicator of regional lymph node status. *FGD3* mRNA expression as a biomarker for an immune response was evaluated by using tumor-infiltrating lymphocyte cells in TCGA breast cancer and Breast International Group 1-98 (BIG 1-98; Letrozole or Tamoxifen in Treating Postmenopausal Women With Breast Cancer) cohorts. *ESR1* transcriptional regulation of *FGD3* mRNA expression in the breast cancer cell line ZR-75-1 was confirmed.

## METHODS

### Cohorts

Detailed description of each cohort is provided in the Data Supplement.

### Survival Analysis

To illustrate the outcome benefit of low versus high expression, the Kaplan-Meier method^13^ was used to estimate the distribution of time-to-disease outcome end points by gene expression status bifurcated on the cohort mean and median (Data Supplement). An overall inference measure was determined by using a Stouffer weighted *Z*-test *P* value^14,15^ to combine probabilities from the cohorts. The Wald-test *P* values from Cox proportional hazards models for the association of cancer outcomes with gene expression as a continuous variable for each cohort were used to determine the Stouffer weighted *Z*-test *P* value. BioJava was used to implement the R survival package^16^ for analysis. Meta-analysis data for combined hazard ratio (HR) and forest plot figures were generated by using MetaXL^17^ (http://www.epigear.com/).

### *FGD3* Expected Tissue Cell Profile and Tumor-Infiltrating Lymphocyte Association

Faciogenital dysplasia 3 (FGD3) protein is found to have high expression in immune response cells according to curated data in 79 human and 61 mouse tissues from the GeneAtlas^18^ using BioGPS.^19^ Tumors with high expression of FGD3 and favorable outcome could indicate an immune response. Tumor-infiltrating lymphocytes (TILs) were called in 389 samples from TCGA breast cancer high-resolution slide images using methods previously defined by the International TIL Working Group 2014.^20^ TILs were called using a similar methodology in 725 samples from the BIG 1-98 DASL cohort, and correlation to FGD3 mRNA expression was determined by using Pearson’s correlation coefficient (*r*).^21^

### FGD3 Protein Expression Analysis

FGD3 protein expression in tumor samples was determined by immunohistochemical (IHC) staining. Breast cancer samples were provided by Avera Cancer Institute, and breast cancer TMAs (BR1504a, BR1505b, HBre-Duc068Bch-01, and BR20837) were purchased from US Biomax (Rockville, MD).

FGD3 protein expression was quantitatively determined in the range of 0 to 4. The US Biomax metadata indicates whether the patient had no regional lymph node metastasis (N0) or degrees of metastasis in regional lymph nodes (N1 to N3). FGD3 expression levels for samples with N0 designation (n = 135) and samples with N1 to N3 designations in metastatic tissue (n = 98) were compared by using an unpaired *t* test. Tissues for matched lymph node metastasis (n = 103) were compared with primary tumor tissues using unpaired *t* test, and figures were generated using GraphPad Prism 7.0. Additional details on analysis can be found in the Data Supplement.

### *FGD3* mRNA Expression Regulated by Estrogen Receptors

Breast cancer cell line ZR-75-1 was grown in RPMI-1640 medium with 10% fetal bovine serum. For the treatment of estrogen, cells were deprived of hormone for 3 days in phenol-free RPMI-1640 medium with 5% charcoal-stripped fetal bovine serum and then treated with either ethanol (vehicle) or 1 nM 17β-estradiol (estrogen) for 16 hours. Reverse transcriptase-quantitative polymerase chain reaction (RT-qPCR) was performed and is described in the Data Supplement.

### mRNA Expression of Genes of Interest With Published Data

The Expression Atlas Web site^22^ was used to query RNA sequencing (RNA-seq) expression levels in breast cancer cell lines. In addition, the Web site was used to search for cancer cell line experiments with a 2.0 or greater fold change for FGD3, and downloaded experimental data were used to illustrate differences in differential expression of the proliferation genes using Prism 7.0.

## RESULTS

### Discovery of FGD3

We undertook an exploratory analysis of 20,464 possible single-gene biomarkers as categorical variables split on the mean in the METABRIC discovery cohort and identified FGD3 mRNA expression as the highest ranked prognostic gene based on the *P* value for overall survival (OS), which we subsequently verified as being prognostic in the METABRIC validation cohort (data not shown). A detailed summary of the genes in this article that were used for Cox models as continuous and categorical variables, Kaplan-Meier figures split on cohort means, and expression profiles can be found in the Data Supplement.

### FGD3 Breast Cancer Prognostic Marker

#### IHC estrogen receptor–positive cohorts.

**Fig 1.**
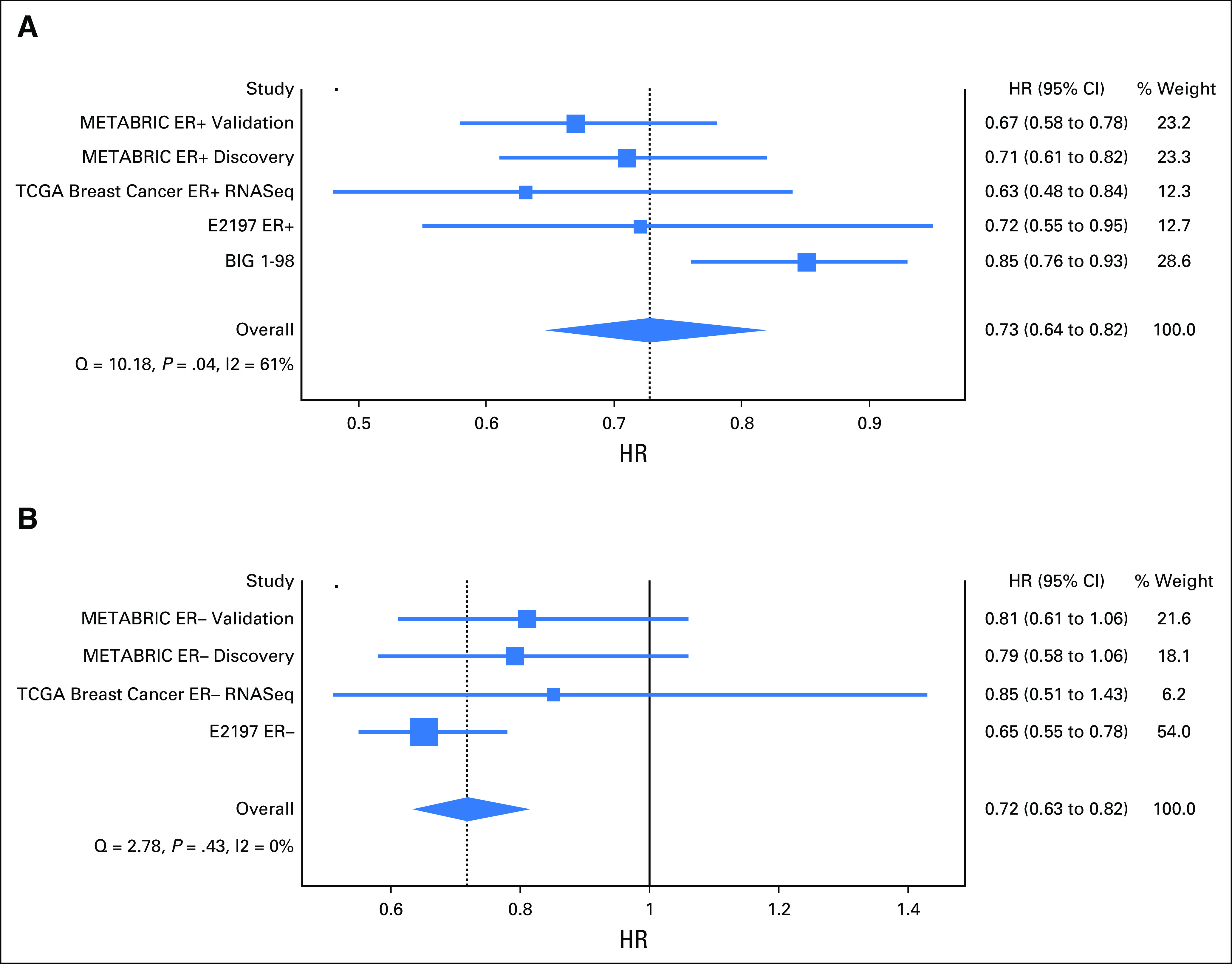
Cox regression analysis for FGD3 messenger RNA (mRNA) expression represented as a *z*-score for overall survival in (A) estrogen receptor (ER)–positive samples determined by immunohistochemistry with an overall hazard ratio (HR) of 0.69 (95% CI, 0.63 to 0.75) and (B) ER-negative samples determined by immunohistochemistry with an overall HR of 0.72 (95% CI, 0.63 to 0.82). Low expression of FGD3 indicates poor outcome in four different cohorts in ER-positive and ER-negative samples. BIG 1-98, Breast International Group 1-98 [trial]; Discovery, discovery cohort; E2197, Combination Chemotherapy in Treating Women With Breast Cancer [trial]; METABRIC, Molecular Taxonomy of Breast Cancer International Consortium; RNA-seq, RNA sequencing; Validation, validation cohort.

The results of the Cox regression analysis of *FGD3* mRNA expression as a continuous variable in five distinct estrogen receptor (ER)–positive breast cancer cohorts are shown in Figure 1A (combined HR, 0.73; 95% CI, 0.64 to 0.82) in which high expression is prognostic of favorable outcome. High expression of *SUSD3* was also prognostic of favorable outcome (HR, 0.72; 95% CI, 0.62 to 0.84; Data Supplement). High expression of *MKI67* is prognostic of poor outcome (HR, 1.19; 95% CI, 0.98 to 1.45) in the five cohorts, but in the E2197 (Combination Chemotherapy in Treating Women With Breast Cancer) ER-positive cohort it had the opposite effect, with an HR of 0.78 (95% CI, 0.62 to 1.00), indicating that low expression is prognostic of poor outcome (Data Supplement). *PCNA* has an HR of 1.06 (95% CI, 0.94 to 1.19) and is prognostic, by virtue of the HR > 1.0, of poor outcome in the METABRIC ER-positive validation cohort (HR, 1.27; 95% CI, 1.11 to 1.45; Data Supplement). *AURKA* has a combined HR of 1.26 (95% CI, 1.03 to 1.54), indicating that high expression is prognostic of poor outcome in METABRIC ER-positive and ER-negative cohorts (Data Supplement). The Stouffer *P* value was used to rank the prognostic significance of each gene in five distinct ER-positive cohorts: *FGD3* (*P* = 3.87^E-14^), *SUSD3* (*P* = 4.15^E-11^), *MKI67* (*P* = 1.06^E-8^), *AURKA* (*P* = 2.61^E-5^), and *PCNA* (*P* = .402).

#### IHC ER-negative cohorts.

In the four ER-negative cohorts (BIG 1-98 was an ER-positive only cohort), the combined meta-analysis for *FGD3* had an HR of 0.72 (95% CI, 0.63 to 0.82) and was prognostic in each cohort (Fig 1B). *SUSD3* had a combined HR of 0.88 (95% CI, 0.74 to 1.05) and no single cohort had a statistically significant *P* value (Data Supplement). *MKI67* had a combined HR of 0.93 (95% CI, 0.83 to 1.04) and no single cohort had a statistically significant *P* value (Data Supplement). *PCNA* had a combined HR of 0.96 (95% CI, 0.80 to 1.15) and was prognostic in the E2197 ER-negative cohort (HR, 0.78; 95% CI, 0.63 to 0.97; Data Supplement). *AURKA* had a combined HR of 1.06 (95% CI, 0.93 to 1.20) and was not prognostic in any individual cohort (Data Supplement). The Stouffer *P* value was used to rank the prognostic significance of each gene in four distinct ER-negative cohorts: *FGD3* (*P* = 3.88^E-5^), *PCNA* (*P* = .063), *SUSD3* (*P* = .319), *MKI67* (*P* = .477), and *PCNA* (*P* = .820).

#### Hungarian Academy of Science breast cancer cohort.

**Fig 2.**
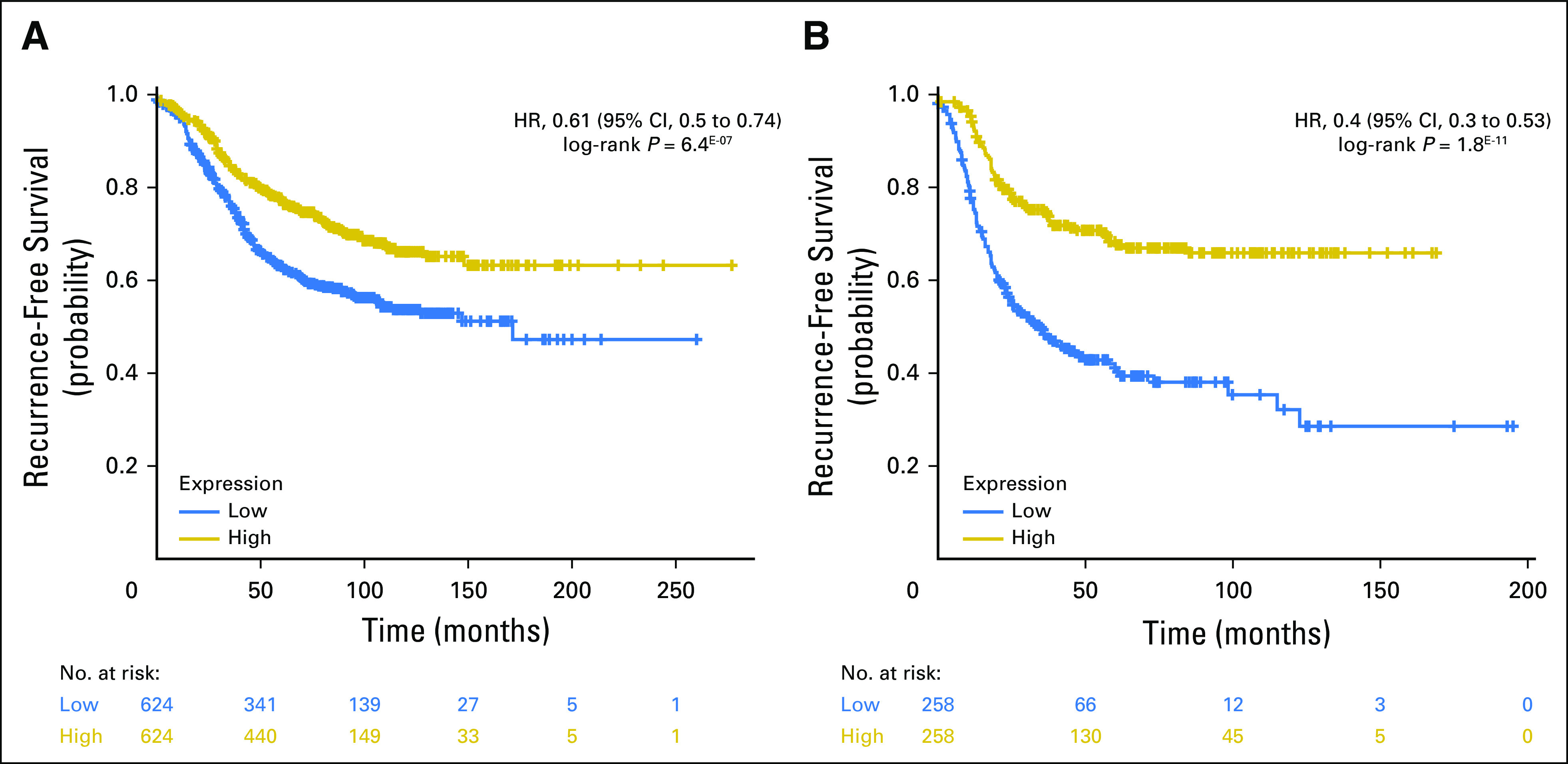
Hungarian Academy of Science breast cancer cohort Kaplan-Meier plots for recurrence-free survival splitting on FGD3 median messenger RNA (mRNA) expression in which estrogen receptor (ER)–positive status is derived from mRNA using the Kaplan-Meier Plotter Web interface to generate graphs. (A) ER-positive (hazard ratio [HR], 0.59; 95% CI, 0.48 to 0.72; *P* = 2.5^E-07^) and (B) ER-negative (HR, 0.37; 95% CI, 0.28 to 0.5; *P* = 5.9^E-12^) data indicate that low expression of FGD3 mRNA is highly prognostic of disease progression.

The Hungarian Academy of Science (HAS) breast cancer cohort represents a collection of all publicly available breast cancer cohorts profiled on the Affymetrix platform. The Kaplan-Meier Plotter Web interface allows selection of HAS cohorts on the basis of ER status using the mRNA expression level. *FGD3* is prognostic in the HAS ER-positive cohort (HR, 0.61; 95% CI, 0.5 to 0.74; *P* = 6.4^E-7^) and in the ER-negative cohort (HR, 0.4; 95% CI, 0.3 to 0.53; *P* = 1.8^E-11^) when split on the median (Fig 2A-B), further supporting that high expression of *FGD3* mRNA denotes favorable outcome. High mRNA expression of *SUSD3* was prognostic of favorable outcome in the HAS ER-positive cohort (HR, 0.61; 95% CI, 0.51 to 0.75; *P* = 6.5^E-7^) and in the ER-negative cohort (HR, 0.54; 95% CI, 0.41 to 0.71; *P* = 6.1^E-6^) split on the median (Data Supplement). Low expression of *MKI67* mRNA was prognostic of good outcome in the HAS ER-positive cohort (HR, 1.4; 95% CI, 1.23 to 1.59; *P* = 2.2^E-7^) split on median but was not prognostic in the HAS ER-negative cohort (*P* = .41; Data Supplement). Low expression of *PCNA* mRNA was prognostic of good outcome in the HAS ER-positive cohort (HR, 1.65; 95% CI, 1.45 to 1.87; *P* = 2.3^E-14^) and the HAS ER-negative cohort (HR, 1.34; 95% CI, 1.08 to 1.65; *P* = .007) split on the median (Data Supplement). Low expression of *AURKA* mRNA was prognostic of good outcome in the HAS ER-positive cohort (HR, 2.1; 95% CI, 1.84-2.4; *P* < 1^E-16^) split on the median and was not prognostic in the HAS ER-negative cohort (*P* = .87; Data Supplement).

#### Prediction analysis of microarray 50 breast cancer prognostic marker.

**Fig 3.**
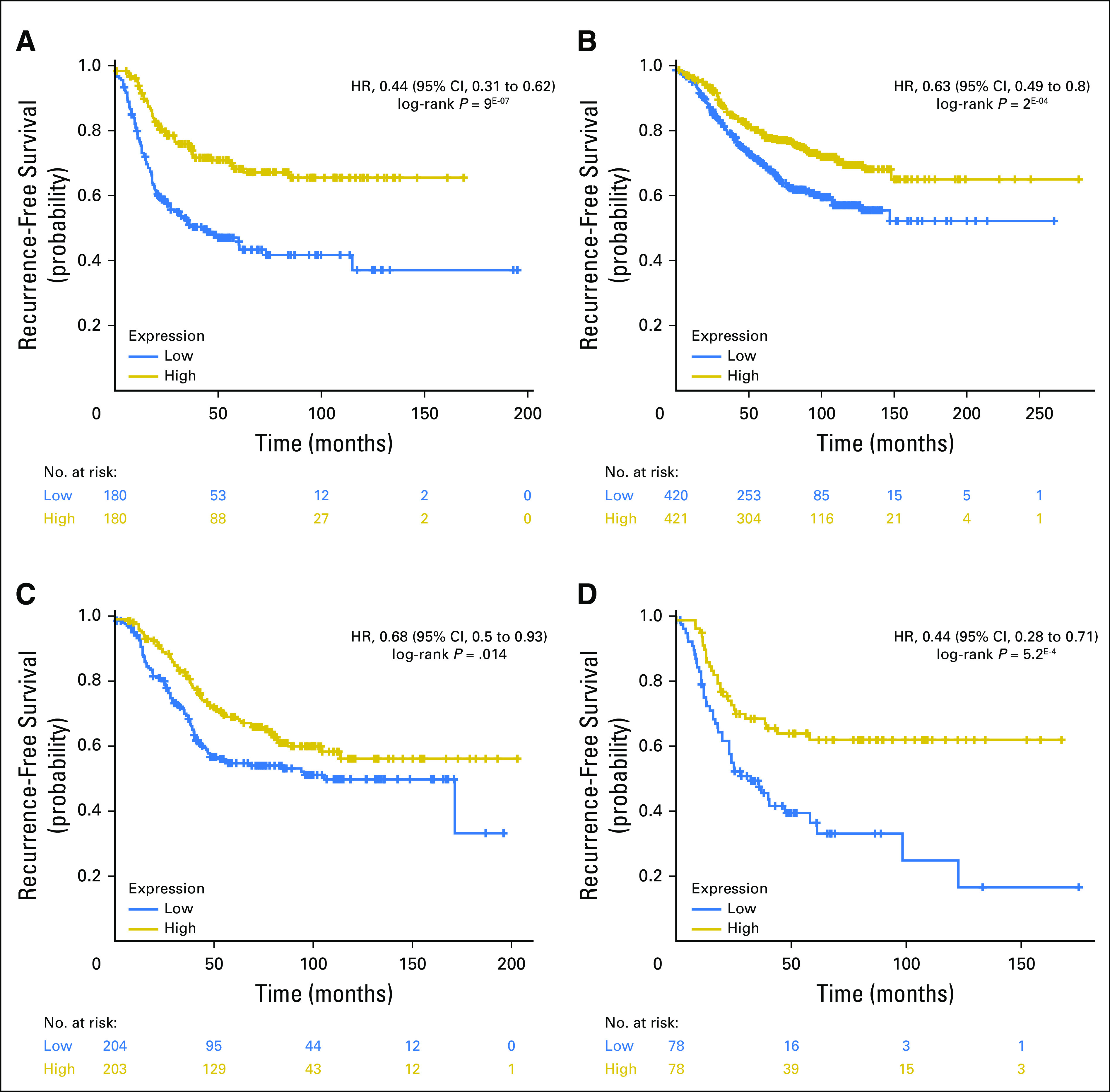
Hungarian Academy of Science breast cancer cohort Kaplan-Meier plot for recurrence-free survival splitting on FGD3 median messenger RNA (mRNA) expression in the prediction analysis of microarray 50 subtype. (A) Basal (hazard ratio [HR], 0.44; 95% CI, 0.31 to 0.62; *P* = 9^E-07^), (B) luminal A (HR, 0.63; 95% CI, 0.49 to 0.8; *P* = 2^E-4^), (C) luminal B (HR, 0.68; 95% CI, 0.5-0.97; *P* = .014), and (D) human epidermal growth factor receptor 2–positive (HR, 0.44; 95% CI, 0.28 to 0.71; *P* = 5.2^E-4^) data indicate that high expression of FGD3 mRNA is highly prognostic of favorable outcome.

To determine whether *FGD3* was a prognostic biomarker in a specific prediction analysis of microarray 50 (PAM50) subtype, the HAS cohort was subdivided via the Web interface for the Kaplan-Meier Plotter. The resulting analysis indicated that FGD3 was prognostic in each subtype: basal HR, 0.44 (95% CI, 0.31 to 0.62; *P* = 9^E-07^), luminal A HR, 0.63 (95% CI, 0.49 to 0.8; *P* = 2^E-4^), luminal B HR, 0.68 (95% CI, 0.5 to 0.97; *P* = .014), and human epidermal growth factor receptor 2 subtype E (HER2-E) HR, 0.44 (95% CI, 0.28 to 0.71; *P* = 5.2^E-4^; Fig 3). *FGD3* mRNA expression was prognostic in each of the HAS breast cancer–defined PAM50 subtypes in which high expression indicated favorable outcome. *SUSD3* mRNA was prognostic in each subtype: basal HR, 0.56 (95% CI, 0.4 to 0.78; *P* = 5.3^E-4^), luminal A HR, 0.66 (95% CI, 0.51 to 0.84; *P* = 8.8^E-4^), luminal B HR, 0.71 (95% CI, 0.52 to 0.96; *P* = .027), and HER2-E HR, 0.57 (95% CI, 0.35 to 0.9; *P* = .015; Data Supplement). *MKI67* was prognostic in luminal A (HR, 1.38; 95% CI, 1.17 to 1.64) and was not prognostic in the other subtypes (Data Supplement). *PCNA* was prognostic in luminal A (HR, 1.64; 95% CI, 1.38 to 1.95; *P* = 1.5^E-8^) and luminal B (HR, 1.52; 95% CI, 1.25 to 1.85; *P* = 1.9^E-5^; Data Supplement). *AURKA* was prognostic in luminal A (HR, 2.3; 95% CI, 1.92 to 2.75; *P* < 1^E-16^) and luminal B (HR, 1.47; 95% CI, 1.21 to 1.78; *P* = 9^E-5^; Data Supplement).

#### HAS lung and ovarian cancer prognostic marker.

*FGD3* high expression of mRNA is prognostic of favorable outcome in the HAS lung cohort^23^ (HR, 0.68; 95% CI, 0.58 to 0.81; *P* = 7.2^E-06^) and the HAS ovarian cohort^24^ (HR, 0.79; 95% CI, 0.63 to 0.99; *P* = .041) when split on the median (Data Supplement). *SUSD3* was prognostic in the HAS ovarian cohort with separation of survival after 4 years (HR, 1.22; 95% CI, 1.01 to 1.48; *P* = .035) in which low expression indicated favorable outcome (Data Supplement). *MKI67* high expression was prognostic of poor outcome in the HAS lung cohort (HR, 1.6; 95% CI, 1.41 to 1.82; *P* = 2.6^E-13^; Data Supplement). *PCNA* was not prognostic in either the HAS lung or ovarian cohorts (Data Supplement). *AURKA* high expression was prognostic for poor outcome in the HAS lung cohort (HR, 1.52; 95% CI, 1.33 to 1.72; *P* = 1.2^E-10^) and the HAS ovarian cohort (HR, 1.19; 95% CI, 1.05 to 1.34; *P* = .0077; Data Supplement).

#### TCGA cancer cohorts prognostic marker.

A complete analysis of FGD3, SUSD3, MKI67, PCNA, and AURKA mRNA expression (*z*- score) as a prognostic biomarker in TCGA RNA-seq cohorts is shown in the Data Supplement. Seven of the 21 TCGA cohorts did not have a single-gene biomarker from the list (FGD3, SUSD3, MKI67, PCNA, AURKA) that was prognostic. FGD3 had a combined HR of 0.91 (95% CI, 0.84 to 0.98). In summary, the statistically significant hits for FGD3 mRNA (*z*-score) as a continuous variable in a Cox regression model included head and neck squamous cell carcinoma (HR, 0.72; 95% CI, 0.63 to 0.81; *P* = 5.99^E-7^), lung adenocarcinoma (HR, 0.78; 95% CI, 0.68 to 0.89; *P* = 3.32^E-4^), cervical squamous cell carcinoma and endocervical adenocarcinoma (HR, 0.69; 95% CI, 0.54 to 0.87; *P* = .002), sarcoma (HR, 0.73; 95% CI, 0.60 to 0.90; *P* = .003), invasive breast carcinoma (HR, 0.82; 95% CI, 0.70 to 0.96; *P* = .015), and urothelial bladder carcinoma (HR, 0.85; 95% CI, 0.73 to 0.99; *P* = .033). By using the biomarker *P* value and the number of samples in each cohort, the Stouffer^25^ *P* value was calculated to rank the prognostic value as a pan-cancer biomarker. AURKA has an overall prognostic Stouffer *P* = 2.85^E-12^; PCNA, *P* = 8.8^E-7^; MKI67, *P* = 3.0^E-6^; FGD3, *P* = 9.04^E-6^; and SUSD3, *P* = 3.21^E-5^. A detailed analysis of each gene in the TCGA cohorts can be found in the Data Supplement.

#### FGD3 expected tissue expression profile.

The mRNA expression profile of a gene can indicate that it might be a biomarker for a particular cell type. *FGD3* is highly expressed in T cells, natural killer cells, myeloid cells, monocytes, and whole blood (Data Supplement). In addition, combined with mRNA expression of FGD3 in cell lines, high expression of *FGD3* could be a positive prognostic biomarker for TILs. By using the TCGA cohort, TILs were called from high-resolution images, and *FGD3* mRNA expression did not correlate with TILs evaluated on hematoxylin and eosin slides defined by using a previously published method^26^ in which TILs represented a pre-existing antitumor T-cell response (unpublished data). In addition, TIL calls from the BIG 1-98 DASL cohort did not correlate with *FGD3* mRNA expression.

**Fig 4.**
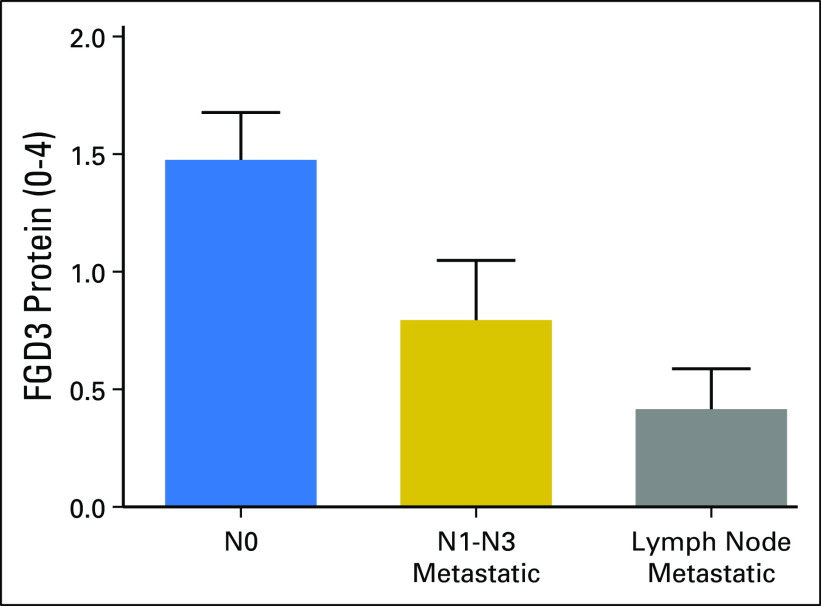
FGD3 protein expression levels determined by using immunohistochemistry in breast cancer tissue microarrays categorized by vendor-supplied metadata. N0, primary tumor with no indication of metastasis to regional lymph nodes (n = 135); N1 to N3, metastatic primary tumor with one to three positive regional lymph nodes (n = 98); and tumor tissue for matched lymph node metastasis (n = 103).

**Fig 5.**
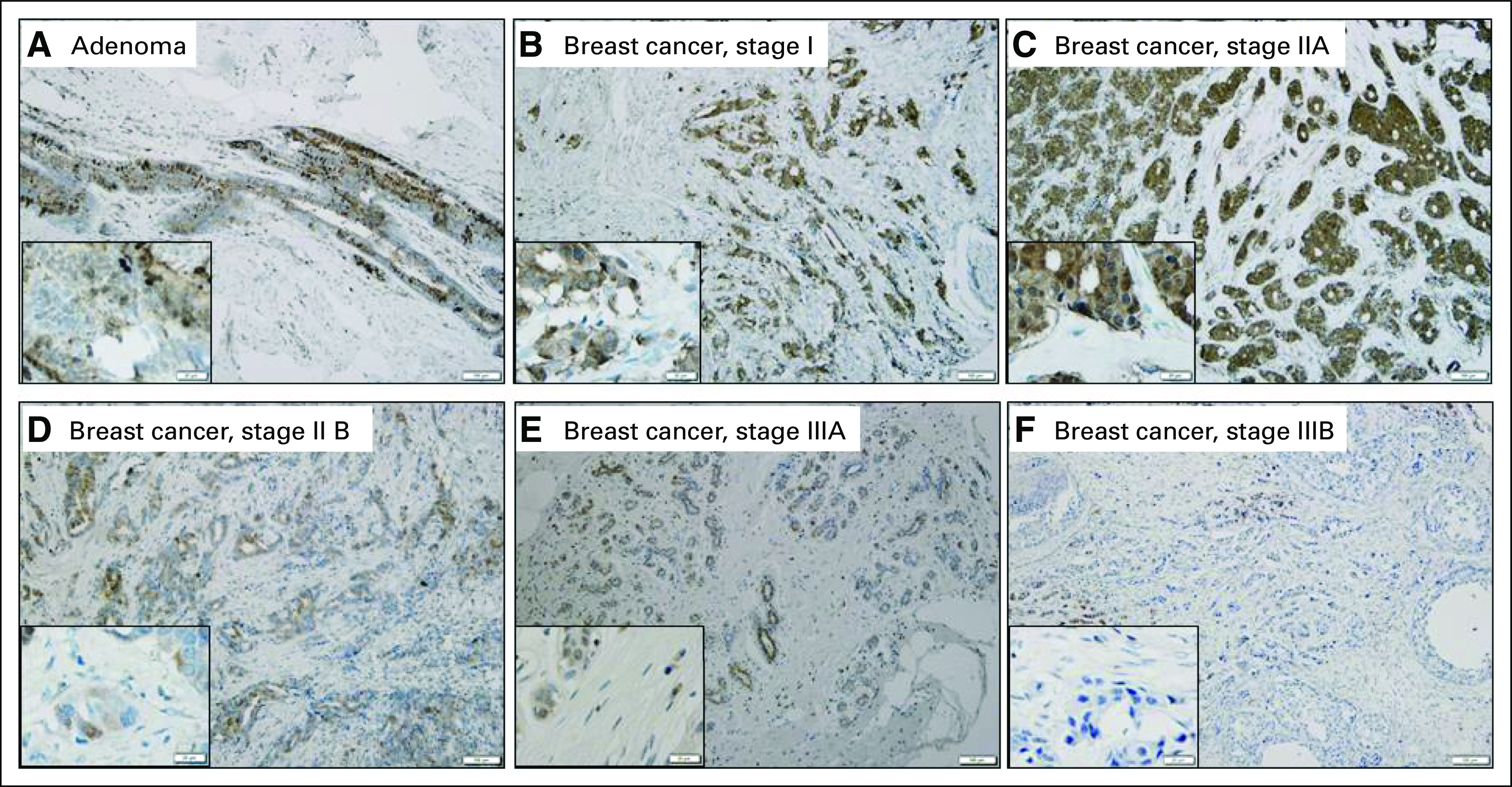
(A-F) Representative findings on immunohistochemical staining of FGD3 (original magnification ×100; insert magnification ×400). (A) Strong FGD3 expression in benign tumor and (B-C) at lower cancer stages I and IIA, (D-E) mild FGD3 expression at stages IIB and IIIA, and (F) weak FGD3 expression at stage IIIB.

To further investigate whether *FGD3* mRNA expression is a feature of the tumor, breast cancer TMAs were purchased from US Biomax, and IHC was used to determine *FGD3* protein expression levels (scored from 0 to 4). *FGD3* protein expression was determined to be a feature of the tumor and was not associated with the presence of lymphocytes. By using metadata provided by US Biomax, *FGD3* protein levels in tumors (n = 135) with no regional lymph node metastasis (N0) were compared with tumors with lymph node metastasis (N1 to N3) and corresponding matched metastatic tissues (Fig 4). An unpaired *t* test comparing N0 with N1 to N3 suggests that lymph node metastasis is associated with lower *FGD3* protein levels (*P* < 1^E-4^). An unpaired *t* test comparing FGD3 protein expression in N1 to N3 primary tumor samples with lymph node metastatic tissue samples suggests that lower *FGD3* protein level indicates metastasis (*P* = .142). Representative images of *FGD3* IHC staining are shown in Figure 5 and the Data Supplement. Benign tumors (Fig 5A) and breast adenocarcinomas in lower stages (Fig 5B-C) showed strong expression of *FGD3*, whereas late-stage breast adenocarcinomas in higher stages (Fig 5D-F) showed mild to weak expression. Stage IIA invasive breast cancer (Data Supplement) showed strong *FGD3* expression compared with its matched metastatic carcinoma (Data Supplement), in which *FGD3* was weakly expressed. These data suggest that *FGD3* protein expression is inversely associated with stage.

Published *ESR1* chromatin immunoprecipitation sequencing data in breast cancer cell lines MCF-7^27^ and ZR-75-1^28^ identified a conserved *ESR1* binding site within the gene locus of *FGD3* (Data Supplement). By using RT-qPCR, we also determined that estradiol stimulation substantially increased the mRNA expression level of *FGD3*, as well as the known *ESR1* targeted gene *TFF1* in the ZR-75-1 cell line (Data Supplement). The Gene Expression Atlas^22^ was queried for cancer cell line experiments with a statistically significant fold change of *FGD3* mRNA with a log fold change greater than 2. In a previously published *ESR1* knockdown experiment in the MCF-7 ER-positive breast cancer cell line, *FGD3* was downregulated by 2.9 and *SUSD3* by 1.3 on a log2 scale (Data Supplement).^29^ cBioPortal was used to query co-expression relationships in the METABRIC cohort, which showed a tendency toward co-occurrence with *P* < .001 and a log odds ratio of 1.52. The TCGA breast cohort showed a tendency toward co-occurrence with *P* < .001 and a log odds ratio of 2.3 in TMA expression data. These data suggest that *ESR1* plays a regulatory role in *FGD3* mRNA expression. Surprisingly, *FGD3* mRNA expression in BT-20, a triple-negative breast cancer cell line, was upregulated to 1.99 and *ESR1* was upregulated to 0.47 on a log2 scale when treated with an *EGFR* inhibitor (Data Supplement).^30^

## DISCUSSION

The exploratory analysis of 20,464 possible single-gene biomarkers in the METABRIC discovery cohort identified FGD3 as a highly prognostic biomarker. Minimal research has focused on it as a possible driver of metastasis in breast cancer, and a pan-cancer analysis in TCGA cohorts found that *FGD3* mRNA was a putative prognostic biomarker in other cancers. This is a significant finding in the TCGA cohorts, considering the median survival time in these cohorts was typically less than 2 years.

*FGD3* has a putative guanine nucleotide exchange factor that targets cell division control protein 42 homolog (CDC42)^31^ and shares high sequence similarity with *FGD1* in their Dbl homology (70%) and pleckstrin homology (60.6%) domains; however, *FGD3* lacks the N-terminal proline-rich domain found in *FGD1*. The proline-rich domain plays a crucial role in the response to extracellular signal-responsive translocation of *FGD1* to the leading-edge membrane in cells facing toward a wound during the wound-healing process.^32^

Through a highly conserved recognized destruction motif (DSGIDS) in both FGD3 and FGD1 as well as in other unrelated proteins including IκBs and β-catenin, downregulation occurs through the ubiquitin/proteasome system via phosphorylation by GSK-3β of the serine residues in the DSGIDS motif.^33^ Thus, both FGD3 and FGD1 intracellular levels are tightly regulated by the same destruction pathway. Functionally, *FGD1* is involved in the regulation of cellular structures required for invadopodia biogenesis and extracellular matrix degradation in an invasive cell model by modulating Cdc42 activation.^34-37^ In addition, mutations in *FGD1* are responsible for the X-linked disorder known as faciogenital dysplasia, and *FGD1* is highly expressed during bone growth and mineralization.^36^

Using the HeLa Tet-Off cell system, Hayakawa et al^31^ showed that notwithstanding their similarity, FGD3 and FGD1 played quite different roles in regulating cellular functions. They found that full-length FGD3 could induce and activate Cdc42. Furthermore, inducible expression of FGD3 resulted in significant morphologic changes that included the formation of broad sheet-like protrusions known as lamellipodia when GTP-bound Cdc42 levels were significantly increased by the inducible expression of FGD3, whereas high expression of *FGD1* led to the formation of filopodia, which are found in migrating cells.^31^ Thus, cell motility seemed to be inversely regulated by FGD3 and FGD1: FGD3 inhibited cell migration and FGD1 stimulated it.

The *FGD3-SUSD3* metagene (these genes share the same chromosomal location directly adjacent to each other at Chr9q22.31) was ranked with the highest concordance index^38^ in the Sage Bionetworks-DREAM Breast Cancer Prognosis Challenge and was a key biomarker presented by the group submitting the winning model.^39^ Using the METABRIC data set,^3^ they determined that the expression value of two genes, *FGD3* and *SUSD3*, was the most prognostic molecular metagene marker associated with a good prognosis, and they referred to it as a protective metagene because it displayed a CI that was significantly less than 0.5. They also validated the poor prognosis associated with low expression of the *FGD3-SUSD3* metagene in the OsloVal data set (described in Liu et al^40^). The prognostic significance of *FGD3* and *SUSD3* as single gene prognostic biomarker using Cox regression models in a large collection of breast cancer cohorts and TCGA cohorts has not been published.

In a follow-up study, the group developed a breast cancer prognostic test, Breast Cancer Attractor Metagenes (BCAM), which had several molecular features, including the breast cancer–specific *FGD3-SUSD3* metagene, as well as tumor size, and number of positive lymph nodes.^41^ Notably, Ou Yang et al^41^ went on to suggest that breast cancer subtype classification as well as hormone receptor and *HER2* status did not add additional prognostic information when expression levels of the *FGD3-SUSD3* metagene and the attractor metagenes were known and taken into consideration.

In a similar manner, *SUSD3* expression was found to be regulated by *ESR1* in MCF-7 cells through direct interaction of E2 with its regulatory region. Experiments in MCF-7 cells further showed that SUSD3 was implicated in E2-mediated cell proliferation, adhesion, and migration.^4^ However, as with FGD3, the role that SUSD3 plays in ER-positive breast cancer has not been fully established.

On the basis of normal tissue expression profiles, *FGD3* is highly expressed in T cells, natural killer cells, monocytes associated with immune response, and myeloid whole blood cells. A characteristic of these cell types is that they are mobile, and evidence from the HeLa Tet-Off wound healing assay suggests that high expression of *FGD3* limits cell mobility.^31^ *FGD3* mRNA expression did not correlate with TILs in the BIG 1-98 DASL and TCGA breast cancer cohorts, suggesting that the prognostic value of *FGD3* is not indicating immune cells in patients’ tumors. IHC data from breast cancer TMAs indicates that *FGD3* protein expression is a feature of the tumor, and low expression indicates a higher chance of cell migration to lymph nodes. The data clearly indicate that *FGD3* may have an important role in metastatic-associated phenotypes. The *FGD3* cell line experiment in this study (Data Supplement) and studies by others^4,29^ suggests that *FGD3* mRNA expression is partially regulated by *ESR1*, an important treatment target in breast cancer, which requires further study.

**Fig 6.**
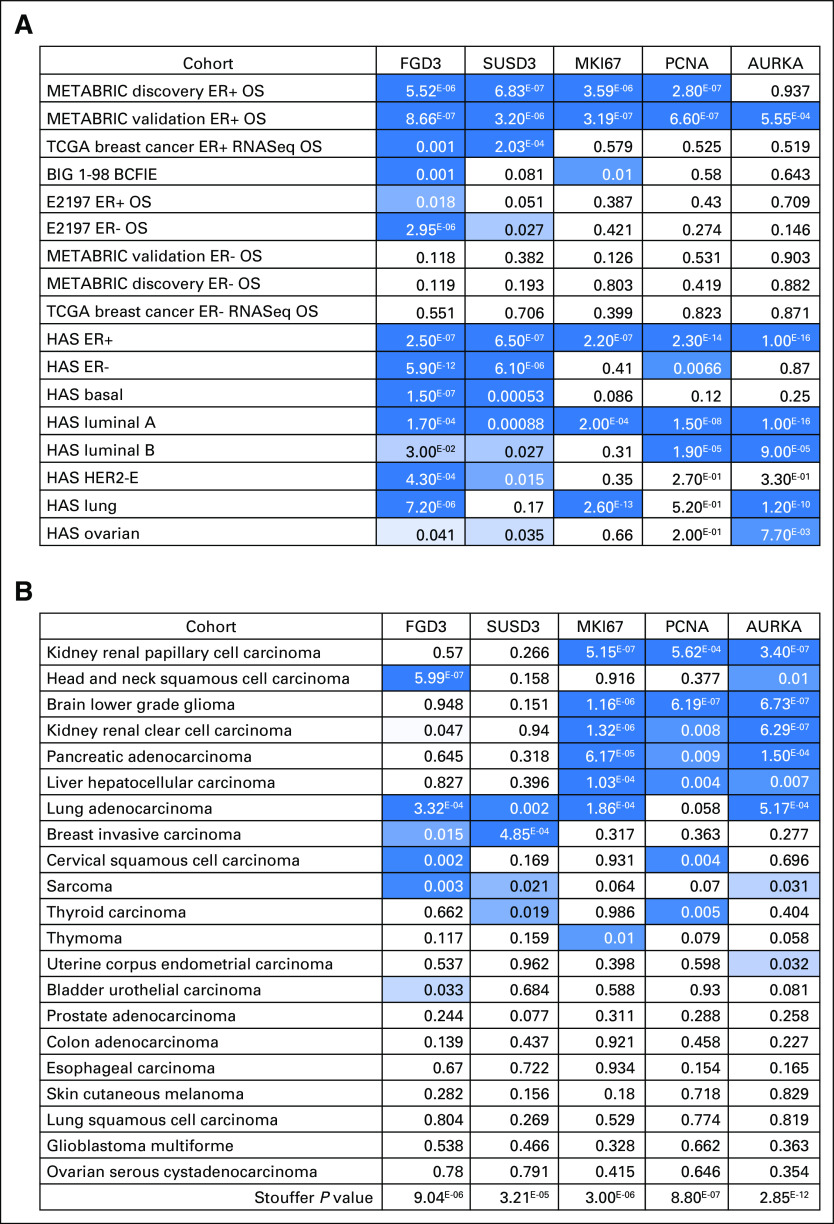
A heat map representation of *P* values for each gene as a continuous variable using Cox regression analysis. (A) FGD3 and SUSD3 are each highly prognostic in breast cancer cohorts. (B) In The Cancer Genome Atlas (TCGA) cohorts, AURKA based on Stouffer *P* value represents the most informative pan-cancer prognostic gene. The seven cohorts at the bottom of the list did not have any of the proliferation genes that were prognostic. BIG 1-98, Breast International Group 1-98 [trial]; E2197, Combination Chemotherapy in Treating Women With Breast Cancer [trial]; ER, estrogen receptor; HAS, Hungarian Academy of Science; HER2-E, human epidermal growth factor receptor 2 subtype E; METABRIC, Molecular Taxonomy of Breast Cancer International Consortium; OS, overall survival; RNA-seq, RNA sequencing.

Given the potential role of *FGD3* in cell migration, it is clearly prognostic in a large collection of breast cancer and other cancer cohorts and has a wide range of treatment options. It has been implicated as a gene that regulates cell migration in the progression of cancer. Comparing the prognostic value of *FGD3* in breast cancer with the prognostic value of genes associated with proliferation such as *MKI67*, *PCNA*, and *AURKA* indicates that *FGD3* may offer superior disease progression metrics in all clinically relevant breast cancer subtypes (Fig 6A). Overall, *AURKA* is the most prognostic gene in the TCGA cancer cohorts in which the median time of 2 years suggests that it is an indicator of early relapse as measured by OS (Fig 6B). FGD3 is prognostic in six TCGA cohorts, and *AURKA* is prognostic in nine TCGA cohorts. *MKI67*, *PCNA*, and *AURKA* are largely prognostic in the same cohorts with renal papillary cell carcinoma, lower-grade brain glioma, renal clear cell carcinoma, pancreatic adenocarcinoma, and lung adenocarcinoma. *FGD3* is uniquely highly prognostic in head and neck squamous cell carcinoma and lung adenocarcinoma. *FGD3* is prognostic in breast invasive carcinoma, cervical squamous cell carcinoma, sarcoma, and bladder urothelial carcinoma. A PubMed search for FGD3 and cancer mentioned in the abstract resulted in one publication.^41^ Repeating the PubMed search for the other proliferation genes resulted in the following publication metrics (MKI67, 92; KI67, 3,104; PCNA, 2,741; and AURKA, 284).

The key differentiator of *FGD3* as a putative biomarker is that high expression indicates favorable outcome; for other established proliferation biomarkers, high expression of *MKI67*, *KI67*, *PCNA*, and *AURKA* are prognostic of poor outcome.

The availability of a growing collection of cancer cohorts with outcome data has allowed for the confirmation of the clinical importance of *FGD3* as a prognostic biomarker and implications that can guide treatment. Given the obvious cohort differences in treatment conditions, patient populations, formalin-fixed paraffin-embedded frozen tissue, and platform differences in Illumina and RNA-seq, *FGD3* represents a robust indicator of outcome in breast cancer as well as other cancers and requires further study.
